# FragKB: Structural and Literature Annotation Resource of Conserved Peptide Fragments and Residues

**DOI:** 10.1371/journal.pone.0009679

**Published:** 2010-03-18

**Authors:** Ashish V. Tendulkar, Martin Krallinger, Victor de la Torre, Gonzalo López, Pramod P. Wangikar, Alfonso Valencia

**Affiliations:** 1 Structural Biology and Biocomputing Programme, Spanish National Cancer Center, Madrid, Spain; 2 Department of Chemical Engineering, Indian Institute of Technology Bombay, Mumbai, India; University of East Piedmont, Italy

## Abstract

**Background:**

FragKB (Fragment Knowledgebase) is a repository of clusters of structurally similar fragments from proteins. Fragments are annotated with information at the level of sequence, structure and function, integrating biological descriptions derived from multiple existing resources and text mining.

**Methodology:**

FragKB contains approximately 400,000 conserved fragments from 4,800 representative proteins from PDB. Literature annotations are extracted from more than 1,700 articles and are available for over 12,000 fragments. The underlying systematic annotation workflow of FragKB ensures efficient update and maintenance of this database. The information in FragKB can be accessed through a web interface that facilitates sequence and structural visualization of fragments together with known literature information on the consequences of specific residue mutations and functional annotations of proteins and fragment clusters. FragKB is accessible online at http://ubio.bioinfo.cnio.es/biotools/fragkb/.

**Significance:**

The information presented in FragKB can be used for modeling protein structures, for designing novel proteins and for functional characterization of related fragments. The current release is focused on functional characterization of proteins through inspection of conservation of the fragments.

## Introduction

Proteins perform key roles in many cellular processes in living organisms. Assigning functions to proteins hold key to understand the molecular mechanism of life. This can be achieved by analyzing proteins sequence or structural properties. Such analysis is carried out at different levels of granularity such as whole structure/sequence or a part of it. The analysis of the whole protein provides general information about its function, while the analysis of local sequence/structure motifs can directly provide clues about functionally important residues in the proteins. Hence we are interested in exploiting local similarity between fragments to derive functional annotations of proteins.

A number of databases of local structural motifs have been constructed by analyzing structural characteristics of protein fragments. These databases are primarily tuned towards discovering sequence-structure relationships in conserved fragments for applications in protein structure prediction. For example, the I-Site library provides sequence structure relationship at the level of fragments [1], while more specialized libraries such as LOOP [2], SLOOP [3] and ArchDB [4] provide sequence-structure relationship in loops. These libraries in general do not provide any information about functional characteristics of conserved fragments. To address this issue, our previous work [5] and a recent publication by Pal and co-workers [6] proposed strategies for adding functional information to structural fragments. Moreover, the analysis of recurrent structural motifs in the context of binding pockets and ligands has been a addressed by Ausiello and co-workers. Such motifs can find similarities in terms of ligand binding even between evolutionary unrelated proteins [7], and resulted in the implementation of the FunClust web server [8]. More traditional approaches for associating functional informations to proteins such as used by the PROSITE [9], PRINTS [10] and Blocks [11] databases extract sequence motifs using multiple sequence alignment approaches in order to infer information on key functional residues. Kasuya and Thornton perform matching of fragments of a given PROSITE pattern using the three dimensional structure to extract corresponding structural motifs [12]. Since sequence motifs are often restricted to a particular family or sub-family, such strategies are unable to capture structural motifs spanning across several families.

We are interested in providing a resource that offers fragment centric fine-grained annotations of proteins. Therefore, we constructed a database of structurally conserved fragments from proteins called FragKB. We characterize each fragment in terms of its sequence conservation, structural similarity and functional descriptions. The functional descriptions are extracted automatically from journal articles using text mining and from manually curated databases.

The information stored in FragKB can be utilized to address various biologically relevant problems. For instance, analysis of sequence-structure relationship in fragment clusters can be exploited to select appropriate candidate fragments for modeling proteins structures. Characterization of structurally conserved fragments derived from evolutionary related proteins may provide clues about important functional regions in the proteins. The structure-function relationship derived from fragment clusters can be used to design novel proteins with altered functional specificity. In this article, we provide a brief description of the database construction workflow, content, its accessibility and interface as well as usability aspects together with a couple of biological example cases.

## Materials and Methods

**Figure 1:**
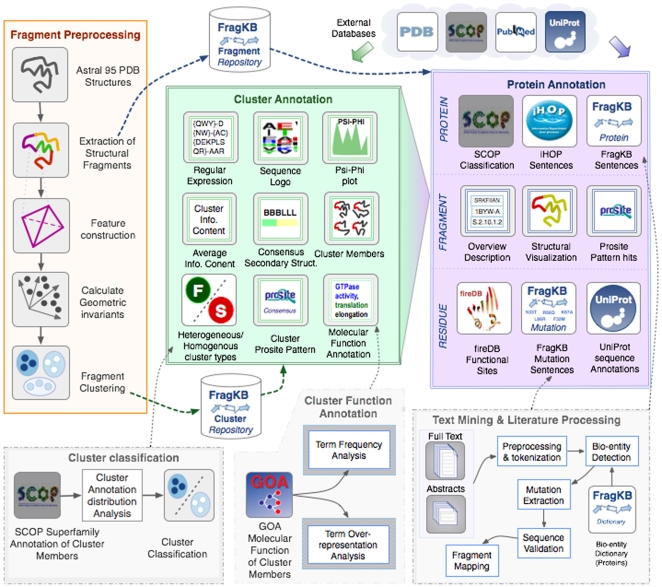
FragKB annotation flowchart. This figure provides an overview of the various steps followed in the annotation workflow followed by FragKB, from the initial generation of structural octapeptide fragments and clusters to the functional annotation at the level of clusters, individual fragments and proteins. A. Fragment Preprocessing: generation of structural fragments and clusters; B. Cluster Annotation: structure, sequence and functional descriptions generated for fragment clusters; C. Protein Annotation: description of fragments at the upper level of the corresponding proteins, the fragment itself and the individual residues; D. Cluster classification method based on SCOP superfamily distribution; E. Cluster Molecular Function Annotation method through Gene Ontology term frequency and over-representation analysis; F. Text Mining and Literature processing for the extraction of protein descriptions and mutations.

The content and FragKB annotation process are illustrated in Figure 1. FragKB stores annotations for two main repositories:

1. **Repository of fragment clusters**: Each cluster contains a set of structurally similar fragments. The clusters are annotated using sequence, structure and function descriptions based on the corresponding properties of its member fragments.
2. **Repository of structural fragments**: The fragments are extracted from protein structures in PDB. Each fragment has a sequence, and three dimensional structure. The functional descriptions are collected at the level of fragment itself along with fine grained annotations at the level of individual residues and coarse annotations at the level of the whole protein.

**Figure 2:**
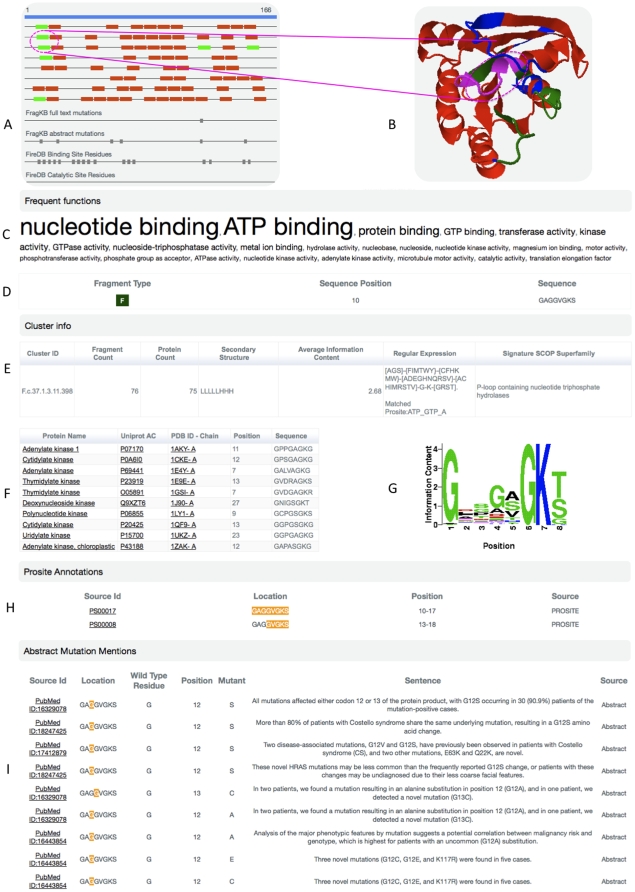
FragKB annotation example: HRAS GTPase. A. Fragment overview of the protein sequence, in green homogeneous fragments can be seen, while heterogeneous fragments are displayed in red. B. Structural visualization of fragment types (green and red) and user selected fragment (pink). C. GO molecular function term frequency analysis of the cluster. D. Fragment summary information and octapeptide sequence. E. Fragment cluster summary showing general cluster descriptions including the cluster regular expression and signature SCOP superfamily association. F. Sample subset of protein cluster list. G. Sequence logo representation showing sequence position versus information content. H. Prosite pattern annotation for the fragment. I. Sample of literature extracted mutation sentence descriptions for G12.

FragKB annotation workflow is based on three broad modules as depicted in Figure 1, namely: (1) Fragment pre-processing and clustering (Figure 1A), (2) Cluster annotations (Figure 1B), (3) Protein annotations (Figure 1C).

### Fragment Preprocessing

The detailed description of the algorithm used for fragment preprocessing and clustering can be found in our earlier publication [5] (Figure 1). Briefly, we extracted fragments from 4,849 representative proteins from the ASTRAL dataset version 1.63 [13]. A sliding window of 8 consecutive amino acid residues was used to extract fragments from a given protein structure, the window slides by one residue at a time. The resulting fragments are made up by eight amino acids and any two given adjacent fragments share an overlap of seven residues. Each fragment is approximated with 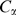 residues [14] and is represented with a set of geometric invariant (GIs) descriptors [15]. The fragments are then subjected to clustering in the space spanned by GIs, which reveals groups of structurally similar fragments.

### Cluster Annotation

The clusters are characterized with various types of annotations based on properties of the member fragments (Figure 1B). At the level of sequence and structure, the clusters are annotated with average 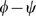 angles, consensus secondary structure and average distance between both the ends of the fragments (in the structure). We derived a sequence logo and average information content using information theoretic analysis of fragment sequences. Note that the information content varies between 0 and 4.2 bits and signifies the overall sequence conservation for a particular cluster, while the sequence logo characterizes position specific residue conservation. Knowledge about conformational preferences and structural regularities, going beyond the primary sequence is captured by the consensus secondary structure and 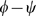 angle plot. General positional preferences of residues for the given cluster are summarized using regular expression sequence pattern. Some of these patterns are known to be functionally relevant and can be found by matching the regular expression with PROSITE patterns. Gene ontology annotations are widely used for providing functional information for biological interpretation of groups of genes and proteins (e.g. gene expression clusters). We have used GO molecular function annotations of the proteins linked to each fragment for functional descriptions of fragment clusters (Figure 1E). The molecular function annotation for a cluster is obtained by scoring each GO term: (1) based on its frequency of occurrence in that cluster and (2) based on its over-representation in that cluster with respect to overall protein annotations contained in FragKB.

We are interested in distinguishing between clusters in FragKB that contain fragments from proteins with a common evolutionary origin as suggested by structural and functional features underlying the SCOP superfamily definition [16] (Figure 1D). With this regard, we categorized our clusters into two types: (i) Homogeneous Clusters and (ii) Heterogeneous Clusters. We used the SCOP classification [16], which is a hierarchical classification of protein structures, for this task. Clusters containing more than 70% for fragments belonging to proteins within the same SCOP superfamily are tagged as signature clusters of that SCOP superfamily and are referred as Homogeneous clusters (F) in FragKB. The remaining clusters are tagged as heterogeneous or structural clusters (S). Note that these clusters contain fragments that are conserved in non-homologous proteins. FragKB contains 2,207 homogeneous clusters and 10,696 heterogeneous clusters. The homogeneous clusters are available for 131 SCOP superfamilies. The homogeneous clusters tend to be small while the heterogeneous clusters are often large in size. The largest cluster is the heterogeneous cluster containing 166,662 alpha-helix fragments. The largest homogeneous cluster contains 306 fragments corresponding to Immunoglobulin SCOP superfamily. The general cluster characteristics are listed in Table 1 arranged according to the cluster type.

**Table 1:** Summary statistics for homogeneous and heterogeneous clusters.

	Homogeneous Clusters	Heterogeneous Clusters
Number of clusters	2,207	10,696
Number of fragments	28,575	437,455
Average Information Content (Bits)	3.24 (+/− 0.51)	1.89 (+/− 0.60)
Average distance between fragment endpoints (Å)	13.50 (+/− 3.86)	13.59 (+/− 3.93)

#### Cluster Nomenclature

Each cluster is labeled using a comprehensive nomenclature based on its characteristics and expressed through a numeric description strategy, with the idea of facilitating a direct interpretation of the underlying cluster properties. These include information content (measured in bits), distance between both the ends of the fragments (measured in Å) and ranking in terms of the number of member fragments. Additionally, SCOP superfamily and the information on cluster groups are included in identifiers of homogeneous and heterogeneous clusters respectively. Note that the identifiers for hetero and homogeneous clusters begin with S and F respectively. For example, F.b.50.1.3.11.7870 is a homogeneous cluster for acid proteases with information content of 3 bits and with an average distances between the two ends of 11 Å, having a ranking of 7,870. The heterogeneous cluster S.25.4.1.9.113 belongs to the 25th super group and 4th subgroup, with an average information content of 1bits together with an end-to-end distance of 9 Å and a rank of 113. This nomenclature system should help the user to quickly grasp the characteristics of a cluster and decide whether it is interestingness based on general cluster properties. Table 1 provides a more general characterization of the clusters in FragKB. Cluster with higher information content may be more interesting to explore since it contains structural fragments with higher sequence conservation possibly due to certain functional specificity, and could be thus prioritized for manual inspection.

#### Protein Annotation

Protein fragment annotations in FragKB are presented at three different layers: (i) Proteins, (ii) Fragments and (iii) Residues. (Figure 1C) Protein level annotations include FragKB protein descriptions systematically extracted from literature, iHOP sentences and SCOP classification. In addition, we provide external links to corresponding UniProt and PDB records. The fragment annotations are extracted from UniProt records and from PROSITE. The amino acid sequence of the fragment is scanned against PROSITE to detect matching patterns for linking PROSITE annotations to the fragments.

#### Protein Annotation at the Residue Level

Residue annotations are derived from UniProt sequence annotations and fireDB, which provides information about functional sites, catalytic and binding sites residues and also the associated ligands in the proteins [17]. For integration of annotations from UniProt, residues are mapped from UniProt sequences to PDB sequences using the SIFT mapping service [18]. Note that this mapping is not available for all the proteins in FragKB. Further, we automatically extract mutation mentions from PubMed abstracts and full text articles (Figure 1F). To associate proteins and mutations co-mentioned within an article, we perform a sequence validation step that involves look up of the wild type residues in protein sequences. We extracted 1,584 mutation mentions from 1,771 full text articles, for 1,273 residues in 3,908 fragments (145 fragments from homogeneous clusters and remaining from heterogeneous clusters). Further, we extracted 3,173 mutations in 2,695 residues belonging to 5,820 fragments (422 fragments from homogeneous clusters and the remaining 5,398 from heterogeneous clusters) from over 9,000 abstracts. This resulting additional information on mutations provides important clues about functional aspects of the corresponding fragments, potential relevance for diseases and also improves human interpretation of biologically relevant aspects through direct literature pointers.

## Results

### Interface and Data Access

FragKB is stored in MySQL relational database and is accessed using PHP application on Apache server on Linux using LAMP stack. The user can search for proteins of interest using one of the following search criteria (i) PDB identifiers, (ii) UniProt accession numbers, (iii) Protein symbol or name, (iv) Organism sources, (v) Gene Ontology terms, (vi) EC number, (vii) Cluster identifier, (viii) SCOP superfamily identifier and (ix) full text search. The full text search allows the user to search by protein names, EC number, Uniprot and SCOP information. The set of conserved fragments in the protein can be visualized using the FragKB fragment browser, which provides an intuitive interface to visualize the fragments in the sequence and also in the three dimensional protein structure. The fragment view also displays mutations extracted by the text mining process. The information about the cluster of the fragment and other fragment annotations are also provided on in the fragment browser.

### Example Annotations

FragKB can be used to obtain information about structurally conserved fragments in the protein of interest. Since FragKB contains literature derived fragment annotation, it enables interpretation of functional significance of a specific conserved fragment. Here, we exemplify the usage of FragKB to find structurally conserved fragments and their functional significance in well-characterized GTPase HRas protein (PDB: 1CTQ).It is known that the small GTPases form an independent superfamily within the larger class of regulatory GTP hydrolases. This superfamily contains proteins that control a vast number of important processes and possess a common, structurally preserved GTP-binding domain. Mutations in these proteins may lead to several chronic diseases in humans. Sequence comparisons of small G proteins from various species have revealed that they are conserved in primary structures at the level of 30–55% similarity [19]. Figure 2 shows a general view of the functional annotation provided for a selected fragment by FragKB. GTPase HRas has 79 conserved fragments (Figure 2A), covering about 75% of the entire sequence. The structure of this protein can be seen in Figure 2B. This protein contains 7 fragments classified into homogeneous clusters that are linked to proteins belonging to the ‘P-loop containing nucleotide triphosphate hydrolases’ SCOP superfamily, as is also the case of GTPase HRas. We observed that members of these particular clusters have been annotated with GO molecular function terms such as *nucleotide binding*, *GTP binding*, *protein binding GTPase activity*, indicating overall similar functional properties shared by these proteins (see Figure 2C). In general, well known functional regions of GTPase HRas are captured by fragments belonging to homogeneous clusters. This is the case of the well known nucleotide-binding region between residues 10–17 (GAGGVGKS), which is captured in the homogeneous cluster F.c.37.1.3.11.398 (Figure 2D and 2E), having 76 fragments from 75 ‘P-loop containing nucleotide triphosphate hydrolases’ proteins. A small subset of them can be seen in Figure 2F. This fragment is located in a region joining a beta strand and an alpha helix, having a compact structure as indicated by the distance between its end points. The cluster has an average information content of 2.68 (bits), which denotes higher conservation in the sequence of these fragments. Particularly G and K are completely conserved at position 6 and 7 respectively, along with higher conservation for G at the first position as depicted by the sequence logo (see Figure 2G). Based on the sequences of fragment members of this cluster the following regular expression is generated,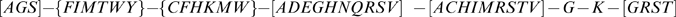 that also matches the ATP_GTP_A PROSITE pattern (Figure 2E and 2H). UniProt documents several natural variants of G12. We are able to extract seven sentences mentioning G12 mutations from the abstracts and substitutions of this residue are considered to be very important oncogenic mutations with clear relevance for cancer. Substitutions of this residue has also additional implications in other human disease conditions such as the Costello Syndrome (Figure 2I). An example of a different type of situation can be the region between residues 57–61 (DTAGQ) known to be part of the catalytic mechanism of GTPase. Despite its specific function the corresponding fragment its detected in a heterogeneous cluster containing other proteins such as interleukin-12 subunit beta, Chitinase A1 and Fe(3+) dicitrate transport protein fecA along with other known GTPase proteins. This cluster has a relatively high information content (1.63 bits) and also several mutation mentions were found for residues belonging to this region. It could be interesting to explore the role of this specialized structural fragment in other non-GTPase proteins. Therefore we analyzed the functional role of the fragment 250–257:A in Chitinase A1 and found that the identified fragment in the cluster is in contact with catalytic residues and with N-acetyl glucosamine oligosaccharide ligand (Figure S1).

## Discussion

FragKB is a database of clusters of structurally conserved fragments annotated with sequence, structure and functionally relevant information. Therefore it complements other fragment libraries that cluster fragments based on sequence similarity, structural similarity or mixture of both.To assist human interpretation of functional roles of the fragments, FragKB provides access to relevant literature information and integrates annotations of proteins, functional sites and sequence patterns through a user-friendly web interface. The integration of text mining approaches in this context, linking structural information to literature descriptions is a novel attempt to assign biological characteristics to proteins. Such systematic information extraction methods are of increasing interest to the biocuration community to improve the manual literature curation workflow process [20]. The underlying systematic annotation workflows followed, facilitates efficient future updates and maintenance of FragKB. Summarizing the biological common properties of a group of bio-entities is still a challenging task, that requires addressing ways for quantifying similarities between functional concepts, another well known different problem currently under intense study. Also with the existing resources, it is not always possible mapping unambiguously between sequences and equivalent protein structures provided by different databases. Furthermore, we plan to collect more annotations, develop additional strategies for function annotation and generate an enhanced cluster representation view.

## Supporting Information

Figure S1 FragKB. Functional implications of heterogeneous clusters. A. Molecular visualization of fragments and functional regions. The green-yellow cartoons represent clustered fragments (Cluster ID: S.1.1.2.12.8046). Labeled residues are implicated in catalysis. Ligands are also highlighted in sticks format. A1. Structural alignment between the Chitinase A1 from *B. circulans* (PDB: 1ITX) and Chitinase A from *S. marcescens* (PDB: 1EDQ), the fragment and functional regions are distant in sequence(B) but close in the 3D structure. A2. human CDC42 homolog (PDB: 2NGR): the fragment comprises the GTPase catalytic motif DTAGQ. A3. Carbonic anhydrase from *P. sativum* (PDB: 1EKJ); the fragment includes the conserved Gln 61; this residue is regarded as catalytic in homologous proteins. B. Functional residue prediction for 1ITX by similarity with 1EDQ. The figure shows the firestar output and the active site of 1ITX (highlighted inside the blue boxes). The fragment (ASGASATY) is highlighted inside the yellow box.
(0.38 MB PDF)
