# Supplementary material for: FragKB: Structural and Literature Annotation Resource of Conserved Peptide Fragments and Residues
Source: PLoS One. 2010 Mar 18;5(3):e9679. doi: 10.1371/journal.pone.0009679 (PMC2841175; doi:10.1371/journal.pone.0009679)
Supplement: Figure S1 — FragKB. Functional implications of heterogeneous clusters. A. Molecular visualization of fragments and functional regions. The green-yellow cartoons represent clustered fragments (Cluster ID: S.1.1.2.12.8046). Labeled residues are implicated in catalysis. Ligands are also highlighted in sticks format. A1. Structural alignment between the Chitinase A1 from B. circulans (PDB: 1ITX) and Chitinase A from S. marcescens (PDB: 1EDQ), the fragment and functional regions are distant in sequence(B) but close in the 3D structure. A2. human CDC42 homolog (PDB: 2NGR): the fragment comprises the GTPase catalytic motif DTAGQ. A3. Carbonic anhydrase from P. sativum (PDB: 1EKJ); the fragment includes the conserved Gln 61; this residue is regarded as catalytic in homologous proteins. B. Functional residue prediction for 1ITX by similarity with 1EDQ. The figure shows the firestar output and the active site of 1ITX (highlighted inside the blue boxes). The fragment (ASGASATY) is highlighted inside the yellow box. (0.38 MB PDF) [file pone.0009679.s001.pdf]

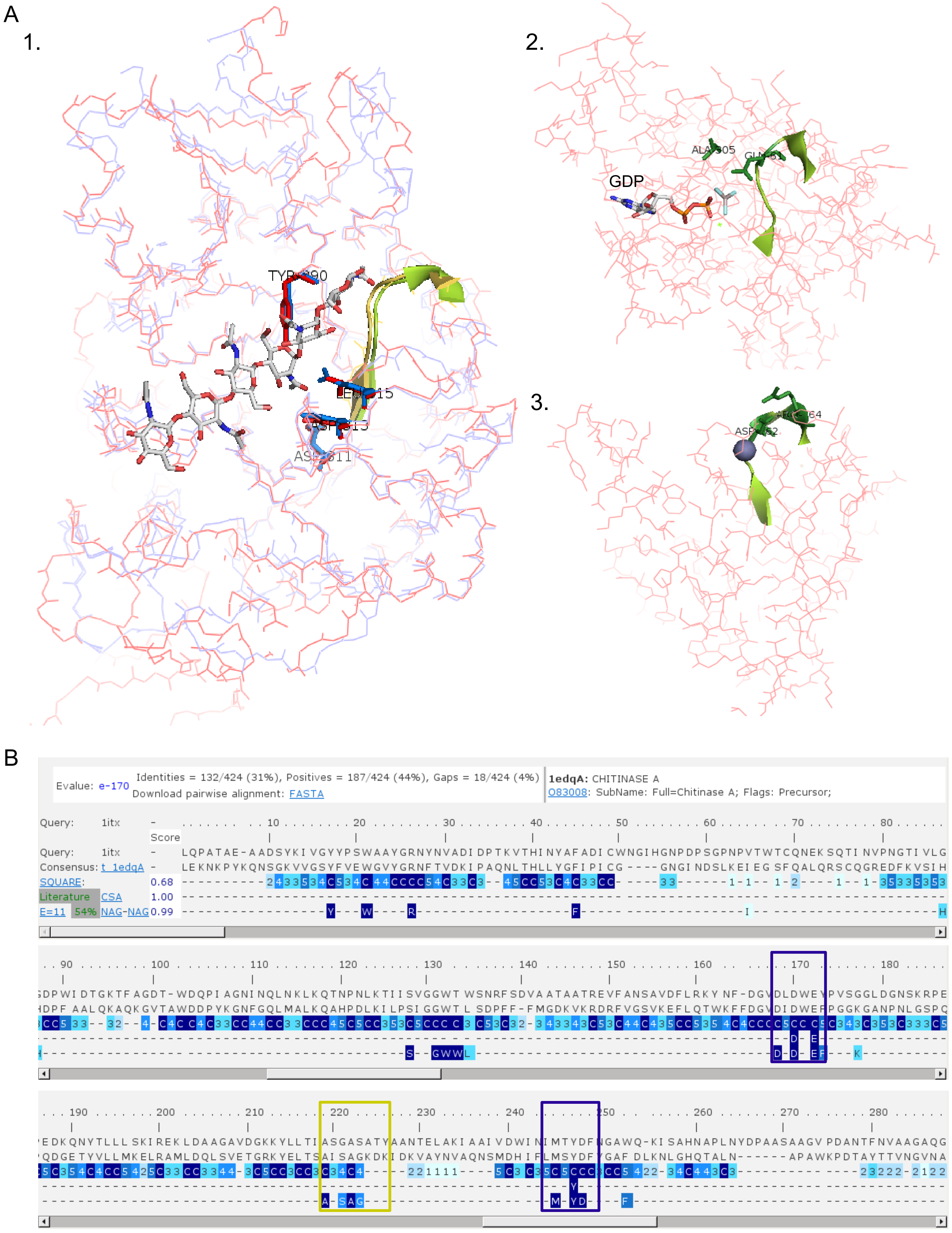

Supplementary figure 1: FragKB. Functional implications of heterogeneous clusters. A. Molecular visualization of fragments and functional regions. The green-yellow cartoons represent clustered fragments (Cluster ID: S.1.1.2.12.8046). Labeled residues are implicated in catalysis. Ligands are also highlighted in sticks format. A1. Structural alignment between the Chitinase A1 from *B. circulans* (PDB: 1ITX) and Chitinase A from *S. marcescens* (PDB: 1EDQ), the fragment and functional regions are distant in sequence(B) but close in the 3D structure. A2. human CDC42 homolog (PDB: 2NGR): the fragment comprises the GTPase catalytic motif DTAGQ. A3. Carbonic anhydrase from *P. sativum* (PDB: 1EKJ), the fragment includes the conserved Gln 61, this residue is regarded as catalytic in homologous proteins. B. Functional residue prediction for 1ITX by similarity with 1EDQ. The figure shows the *firestar* output and the active site of 1ITX (highlighted inside the blue boxes). The fragment (ASGASATY) is highlighted inside the yellow box.
